# Hypochlorous acid solution serves as a potential anti-biofilm therapy for periodontitis *via* targeting quorum sensing of periodontal pathogens

**DOI:** 10.1080/20002297.2025.2557959

**Published:** 2025-09-12

**Authors:** Xuerong Lv, Xiang Han, Yiyang Yang, Yuzhuo Ma, Yue Wang, Kewei Zhang, Feiyang Wang, Chen Yang, Ke Yan, Xiaoqian Wang

**Affiliations:** a The Affiliated Stomatological Hospital of Nanjing Medical University, Nanjing, People's Republic of China; b State Key Laboratory Cultivation Base of Research, Prevention and Treatment for Oral Diseases, Nanjing, People's Republic of China; c Jiangsu Province Engineering Research Center of Stomatological Translational Medicine, Nanjing, People's Republic of China

**Keywords:** Periodontal disease(s)/periodontitis, biofilm(s), quorum sensing, microbiology, biocompatibility, bone loss

## Abstract

**Backgroud:**

Hypochlorous acid solution (HAS), a novel bio-friendly antimicrobial, has garnered attention for its antimicrobial activity, while less is known about its antibiofilm effects on periodontal pathogenic biofilms and the underlying mechanisms.

**Objective:**

This study aimed to explore HAS's antibiofilm effect on periodontal pathogenic biofilms and the potential mechanisms.

**Design:**

*In vitro*, the minimum inhibitory concentration (MIC) of HAS was determined by microdilution method. Alterations in biofilms were analysed using crystal violet (CV) staining, MTT assay and microscopic imaging techniques. The biocompatibility of HAS was assessed *via* CCK-8 and scratch assays. The regulatory mechanism of HAS within biofilms were investigated using bioluminescence assays, reactive oxygen species (ROS) detection and RT‒qPCR. *In vivo*, rat periodontitis models were established. Imaging and histological techniques were employed to evaluate the inhibitory effects of HAS on alveolar bone resorption and inflammatory cytokines.

**Results:**

Compared to 0.25% NaClO solution, it exhibited better biocompatibility. HAS downregulated biofilmvirulence factors and upregulated oxidative stress response-related genes, suggesting that inducing ROS production is a crucial mechanism of HAS in biofilm inhibition. Furthermore, HAS significantly inhibited autoinducer-2 (AI-2) activity and downregulated the QS-related genes. *In vivo*, HAS significantly reduced bone resorption and periodontal inflammation.

**Conclusions:**

Given HAS's accessibility, excellent biocompatibility, and outstanding antibiofilm properties, it may offer a safe antibiofilm approach for clinical periodontal therapy, effectively removing biofilms in areas inaccessible to instrumental therapy and persistent biofilms.

## Introduction

Periodontitis, a chronic inflammatory and immune-reactive disease affecting periodontal supporting tissues, is caused mainly by the imbalances between subgingival bacterial plaque and host immune defence. Such imbalances lead to periodontal tissue destruction and even tooth loss [1]. Periodontal infections enable pathogenic bacteria and their toxic products to penetrate deep periodontal tissues and spread throughout the whole body to cause systemic diseases [2,3].

The current consensus is that maintaining periodontal health requires preserving oral biofilm balance [4]. Compared to planktonic bacteria, biofilms exhibit higher resistance to antibacterial drugs [5]. Consequently, strategies targeting the elimination of biofilm are emerging as promising approaches for controlling periodontal infection.

 Chlorhexidine (CHX) and sodium hypochlorite (NaClO) are commonly used in dental treatment. Previous studies have shown that 0.25% NaClO solution as the periodontal irrigation significantly reduces plaque levels and bleeding on probing in periodontal treatment [6,7]. However, conventional antiseptics like CHX and NaClO exhibit significant cytotoxicity toward periodontal cells. For instance, CHX suppresses fibroblast proliferation and migration at sublethal concentrations [8], while NaClO induces apoptosis in gingival keratinocytes [9]. These adverse effects impede periodontal tissue health and regeneration. Thus, it is urgent to explore new biocides with fewer complications and side effects.

 Hypochlorous acid solution (HAS) is a chlorine-based disinfectant produced by the electrolysis of chlorinated water. It works swiftly, sterilises efficiently, is easily accessible for large-scale use and relatively economical. Studies have shown that, compared to other disinfectants, HAS has better biocompatibility, does not interfere with wound healing and is less corrosive to metals [10–13]. In dentistry, HAS has been introduced as a disinfectant in dental chair units (DCUs), root canal rinses and other treatments [14–16]. Neutral electrolysing water reduces the expression of quorum-sensing (QS) genes in Gram-negative *Helicobacter pylori* (*H. pylori*), thereby inhibiting biofilm formation [17]. Based on the strong lethality of HOCl against Gram-negative bacteria, we considered it a suitable candidate to destroy recalcitrant biofilms composed of periodontal pathogens, the majority of which are Gram-negative.

While most current studies on HAS focus on its sterilising effect, periodontitis treatment requires not only antibiofilm abilities but also biofriendly properties, such as minimal oral mucosa irritation and no interference with wound healing. Therefore, this study investigated HAS as an antibiofilm therapy for periodontal biofilms. Its efficacy and biocompatibility were evaluated through direct application to biofilms and periodontal cells, alongside localised administration in rat periodontitis models. The aim of this study is to explore HAS's antibiofilm effects and mechanisms both *in vivo* and *in vitro* and to examine its scientific validity and feasibility as an adjunctive periodontal therapeutic agent.

## Materials and methods

### Bacteria culture

There were four species of bacteria, including *Porphyromonas gingivalis* (*P. gingivalis*, ATCC 33277), *Fusobacterium nucleatum* (*F. nucleatum*, ATCC 25586), *Prevotella intermedia* (*P. intermedia*, ATCC 25611) and *Enterococcus faecalis* (*E. faecalis,* ATCC 29212). Brain–heart infusion broth (BHI) was supplemented with 5 μg/mL hemin and 1 μg/mL vitamin K1 to prepare brain–heart infusion-supplemented (BHIS) liquid media [18]. The solid media of BHI blood agar was obtained after supplementing the liquid media with 20 mg/mL brain-heart infusion agar (Oxoid Inc., Ogdensburg, NY, USA) and 50 μL/mL sterile defibrinated sheep blood.

### Solution preparation

Hypochlorous acid stock solutions (500 and 2000 mg/L, Flashwater Biological Technology, Nantong, China) were diluted with sterilised bacterial or cell culture media to different concentrations (pH = 7.1 ± 0.2). The commonly used oral antibacterial solution, 0.25% NaClO solution (Longly Biotechnology, Wuhan, China), was prepared as a positive control.

### Construction of biofilms

Two biofilm models, namely, multi-species (*P. gingivalis*, *F. nucleatum*, *P. intermedia* and *E. faecalis*) biofilms and single-species (*P. gingivalis*) biofilms were established in 96-well plates. Using the turbidimetric method, the concentrations of *P. gingivalis*, *F. nucleatum* and *P. intermedia* were adjusted to 10^8^ CFU/mL, and the concentration of *E. faecalis* was adjusted to 10^7^ CFU/mL [18]. These 4 strains of bacteria were mixed in the ratio of 1:1:1:1, then the mixed bacterial suspensions were cultured for 48 h to construct the multi-species biofilms [19–21]. The single-species biofilms were formed with 10^8^ CFU/mL *P. gingivalis* [22].

### Planktonic bactericidal assays

The minimum inhibitory concentration (MIC) value of HAS was obtained by the broth microdilution method. Bacterial cultures treated with HAS at different concentrations (0, 6.5, 12.5, 25, 50, 100, 200, 400 and 800 mg/L) were added to 96-well plates. The 96-well plates were incubated at 37 °C in a bacterial incubator for 48 h, after which the MIC of the test compound was determined as the lowest drug concentration showing no visible bacterial growth, with parallel quantification of bacterial density through optical density measurements at 600 nm (OD_600_) using a microplate reader.

### Biofilm treatment

In biofilm formation experiments, negative control groups (bacterial suspensions treated with PBS) and experimental groups (treated with HAS at final concentrations of 25, 50, 100 and 200 mg/L) were anaerobically incubated at 37 °C for 48 h in 96-well plates. For biofilm dispersal assays, mature biofilms were initially established, after which the original medium was aspirated and replaced with either PBS (negative control groups) or HAS at identical concentrations (25, 50, 100 and 200 mg/L), followed by 24-h of reincubation in fresh BHI medium under anaerobic conditions at 37 °C.

### Biofilm biomass and biofilm metabolic activity

Biofilm biomass was evaluated by crystal violet (CV) staining [23]. The metabolic activities of the collected biofilms were measured by 3-(4,5-dimethylthiazol-2-thiazolyl)-2,5-diphenyl tetrazolium bromide (MTT) assay [24].

### Visualisation of biofilms

Scanning electron microscope (SEM) was used to observe the biofilms. Confocal laser scanning microscope (CLSM) was used to observe live and dead bacteria in biofilms, and the working solution was prepared according to the instructions of LIVE/DEAD™ BacLight™ Bacterial Viability Kit (Thermo Fisher, MA, USA).

### Biocompatibility of HAS

The human oral keratinocyte (HOK) cells line (Jiangsu Province Key Laboratory of Oral Diseases) and human periodontal ligament fibroblasts (hPLFs) were used in this study. The detailed methods are given in Supplementary information.

The migration test was conducted using HOK. The cells in the experimental groups were treated with HAS (final concentrations: 0, 25, 50, 100 and 200 mg/L), in the positive group were treated with 0.25% NaClO solution, and the culture time was 10 min, 1, 3, 6, 12 and 24 h. The migration rate of the cells was measured using ImageJ software. The formula for the calculation of cell migration rate (CMR) is: CMR (%) = (initial scratch area − scratch area after treatment)/initial scratch area × 100%.

The cytotoxicity test was conducted using hPLFs and HOK Negative control wells (baseline control: cells cultured in complete medium without any treatment, 100% viability reference), blank control wells (containing only cell culture medium) and positive control (PC) wells (cells treated with 0.25% NaClO solution) were established simultaneously. The cytotoxicity was determined by using the cell counting kit-8 (CCK-8, Beyotime, Shanghai, China) assay. The formula for the calculation of relative growth rate (RGR) is: RGR (%) = (OD_450_ of test group − OD_450_ of blank control)/(OD_450_ of negative control − OD_450_ of blank control) × 100%.

### RNA extraction, reverse transcription and quantitative real-time PCR (RT-qPCR)

The biofilms prepared according to the above method were harvested and resuspended by Bacteria RNA Enhancement Reagent. Total RNA extraction and reverse transcription were performed using the kit (Vazyme, Nanjing, China), following the manufacturer's instructions. Purified RNA was dissolved in 20 μL of DEPC-treated water and stored at −80 °C until cDNA labelling. cDNA was reverse transcribed using a cDNA synthesis kit (Vazyme) to generate cDNA. cDNA samples were stored at −20 °C until further use.

 The cDNA and negative control were amplified by the Roche LightCycler 480 real-time PCR detection system (Roche, Basel, Switzerland). The reaction mixture (20 μL) contained 10 μL of SYBR Premix Ex Taq II (2×; Vazyme), 5 μL of template cDNA, 0.4 μL of forward and reverse PCR primers and 4.2 μL of sterile distilled water. The qRT‒PCR conditions included initial denaturation at 98 °C for 5 min, followed by a 40-cycle amplification consisting of denaturation at 98 °C for 15 s, annealing at 60 °C for 15 s and extension at 72 °C for 30 s. The genes are listed in Appendix Table 1 [25–30].

**Table 1.** MIC of HAS on planktonic cell growth (mg/L).

Bacteria	*F. nucleatum*	*E. faecalis*	*P. gingivalis*	*P. intermedia*
MIC (mg/L)	200	200	400	400

### Quorum-sensing autoinducer-2 (AI-2) inhibition assay

The inhibitory effect of QS AI-2 was determined by bioluminescence using *Vibrio harveyi* (*V. harveyi*) BB170 as a biosensor and *P. gingivalis* supernatant as a measure of AI-2 activity [31]. *P. gingivalis* was cultured overnight at 37 °C under anaerobic conditions with HAS at different concentrations (0, 25, 50, 100 and 200 mg/L). A *P. gingivalis* supernatant was obtained by centrifugation at 10000 × g for 10 min. AI-2 in the supernatant was detected using the biosensor *V. harveyi* BB170 [32]. The *V. harveyi* BB170 strain was grown at 30 °C in autoinducer bioassay (AB) medium overnight and diluted 1:5000 in fresh AB medium. Then, 180 µL of the mixture and 20 µL of the supernatant were added to 96-well white plates. The bioluminescence of AI-2 biosynthesis intensity was measured.

### Intracellular reactive oxygen species content (ROS)

The ROS level was determined using a reactive oxygen detection kit (Nanjing Jiancheng Bioengineering Institute, Nanjing, China) by fluorescence probe (DCFH-DA, 2,7-dichlorodi-hydrofluorescein diacetate). The samples were centrifuged (2000 rpm for 5 min), washed once, resuspended in 10 μM of DCFH-DA working solution and incubated for 40 min at 37 °C in the dark. The ROS content was measured using a fluorescent microplate meter (Variskon flash, Thermo Fisher Scientific, China) at an excitation wavelength of 502 nm and an emission wavelength of 530 nm.

### *In vivo* assessment of bone resorption and periodontal inflammation, and animal model construction

All animal-related procedures complied with the Guidelines for the Care and Use of Laboratory Animals of Nanjing Medical University and were approved by the ethics committee of Nanjing Medical University (IACUC-2411066). All animal-related procedures in this study complied with the ARRIVE 2.0 guidelines. Male Sprague–Dawley (SD) rats were fed regular food and sterile water throughout the experiment.

 After 1 week of acclimatisation feeding, 16 rats were randomly divided into 4 groups: (A) no ligation (NC group), (B) ligation + 100 mg/L HAS, (C) ligation + 0.25% NaClO solution and (D) periodontitis group, ligation only without treatment (PC group). The rats in Groups B, C and D were anesthetised systemically, after which the bilateral maxillary second molars were ligated with 5−0 silk sutures (checked twice weekly and re-ligated if detached). Concurrently, 200 µL of *P. gingivalis* suspension (10⁸ CFU/mL in carboxymethylcellulose sodium) was inoculated into the gingival sulci of the second molars, with biweekly monitoring for 21 days to establish a standardised periodontitis model for subsequent *in vivo* investigations. Upon successful establishment of the periodontitis model after 21 days, the ligature wires were removed from all the groups. The test solutions were administered *via* gingival sulcus injection three times per week, and after 4 weeks of treatment, all the rats were euthanised, and tissue samples were collected for subsequent experiments.

### Micro-CT analysis

Three-dimensional images were reconstructed using Materialise Mimics 21.0 software. The alveolar bone loss (ABL) was measured in the rats by measuring the distance between the alveolar bone crest (ABC) and cementoenamel junction (CEJ) of the maxillary first and second molars. The bone microarchitecture parameters were analysed and calculated, including bone volume fraction (BV/TV), trabecular thickness (Tb. Th), trabecular number (Tb. N) and trabecular separation (Tb. Sp).

### Histological analysis

The fixed rat maxillaries were decalcified with disodium ethylenediamine tetraacetate (EDTA) on a thermostatic oscillator and then dehydrated in a dehydrator with a range of ethanol solutions. Four-micrometre-thick paraffin sections were prepared and stained with haematoxylin and eosin (H&E) for structural analysis.

 Immunohistochemistry (IHC) staining was performed to evaluate periodontal tissue inflammation following the manufacturer's instructions. IHC analysis was conducted using anti-TNF-α (GB11188, Servicebio) and anti-IL-1β (GB11113, Servicebio), and the results of staining were quantified using ImageJ software.

### Statistical analysis

All the statistical analyses of the data were performed using GraphPad Prism 9.0. Analysis of variance (ANOVA) was used to analyse various factors. In addition, Tukey's test assessed differences between means, with *p* < 0.05 representing significant differences. Significance levels: *p < 0.05, **p < 0.01, ***p < 0.001 and ****p < 0.0001.

## Results

### HAS inhibited the growth of planktonic periodontal pathogenic bacteria

**Figure 1.**
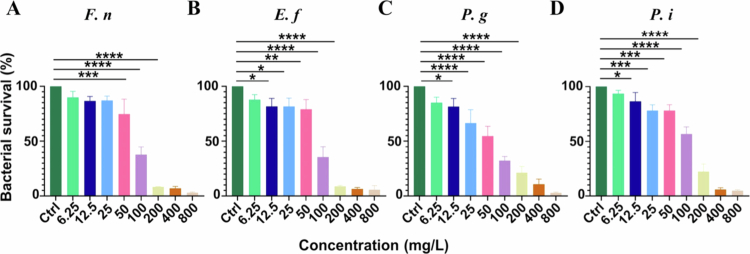
Effect of different concentrations (0, 6.25, 12.5, 25, 50, 100, 200, 400 and 800 mg/L) of HAS on the survival rates of the planktonic (A) *F. nucleatum*, (B) *E. faecalis*, (C) *P. gingivalis* and (D) *P. intermedia*. *p < 0.05, **p < 0.01, ***p < 0.001 and ****p < 0.0001.

As shown in Table 1, the MIC values of HAS against *F. nucleatum* and *E. faecalis* were 200 mg/L, while those against *P. gingivalis* and *P. intermedia* were 400 mg/L. The survival rates of all four planktonic bacteria exhibited a dose-dependent decline with increasing HAS concentration (Figure 1). Notably, HAS at concentrations above 50 mg/L significantly inhibited bacterial growth (*p* < 0.05). Based on these MIC values, HAS concentrations of 25, 50, 100 and 200 mg/L were selected for subsequent studies on the effects against *in vitro* periodontal pathogenic biofilms.

### HAS inhibited periodontal pathogenic biofilms *in vitro*

**Figure 2.**
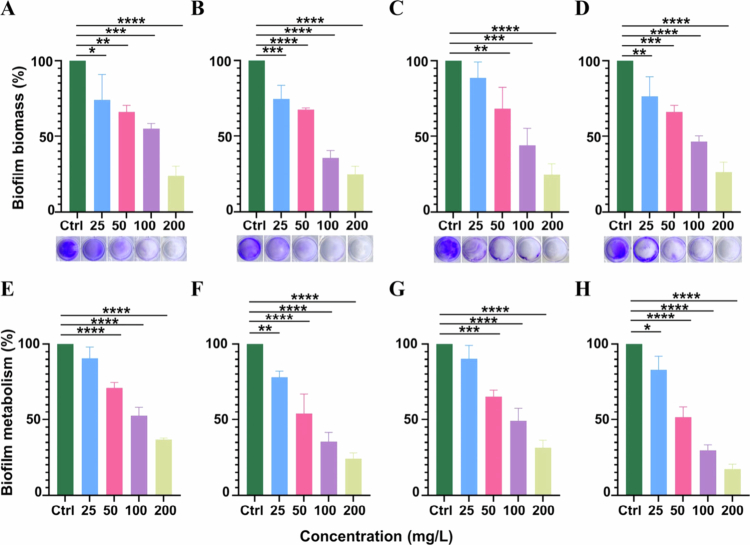
Effect of HAS on biomass and metabolic activity of multi- and single-species biofilms. (A) Biomass of multi-species biofilm formation. (B) Biomass of single-species biofilm formation. (C) Biomass of dispersal of preformed multi-species biofilm. (D) Biomass of dispersal of preformed single-species biofilm. (E) Metabolic activity of multi-species biofilm formation. (F) Metabolic activity of single-species biofilm formation. (G) Metabolic activity of dispersal of preformed multi-species biofilm. (H) Metabolic activity of dispersal of preformed single-species biofilm. *p < 0.05, **p < 0.01, ***p < 0.001 and ****p < 0.0001.

CV staining was employed to assess biofilm growth. Compared to the NC group, in the biofilm formation assay (Figure 2A,B), 25 mg/L HAS reduced the multi- and single-species biofilm biomass by 17.66 ± 16.97 and 27.72 ± 8.86%, respectively. In the biofilm dispersal assay (Figure 2C,D), 50 mg/L HAS decreased preformed multi-species biofilm biomass by 28.66 ± 15.42%, while HAS (>25 mg/L) significantly reduced *P. gingivalis* biofilm biomass by 17.66 ± 13.25% (*p* < 0.05).

An MTT assay was used to evaluate the impact of HAS on the metabolic activity of biofilms. Compared to the NC group, in the biofilm formation assay (Figure 2E,F), 50 mg/L HAS reduced the metabolic activity of multi-species biofilms by 27.53 ± 3.65%, while 25 mg/L HAS significantly decreased the metabolic activity of *P. gingivalis* biofilms by 20.31 ± 4.01% (*p* < 0.01). In the biofilm dispersal assay (Figure 2G,H), 50 mg/L HAS diminished the metabolic activity of preformed multi-species biofilms by 35.90 ± 4.24%, and HAS (>25 mg/L) caused a reduction in *P. gingivalis* biofilm metabolic activity.

**Figure 3.**
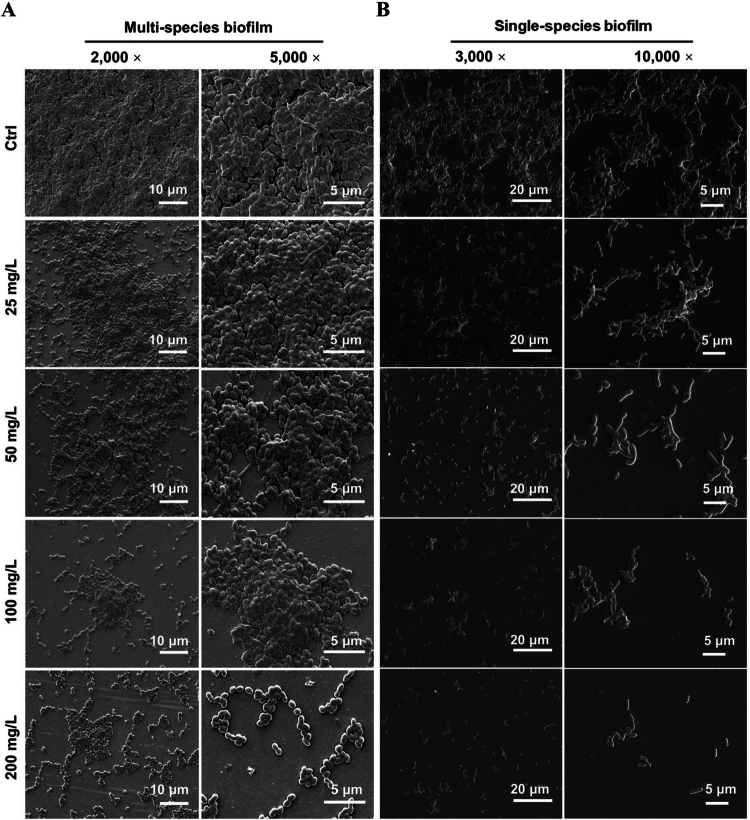
SEM images of HAS' dispersion on preformed multi- and single-species biofilms treated with different concentrations of HAS.

To further validate the anti-biofilm efficacy of HAS, SEM was used to characterise preformed biofilms treated with HAS. As the concentration increased, the biofilm structure became substantially loosened with reduced intercellular connections and partial bacterial detachment (Figure 3). Notably, 200 mg/L HAS nearly degraded the biofilm architecture, resulting in drastic bacterial depletion and rendering the three-dimensional architecture nearly indistinguishable.

**Figure 4.**
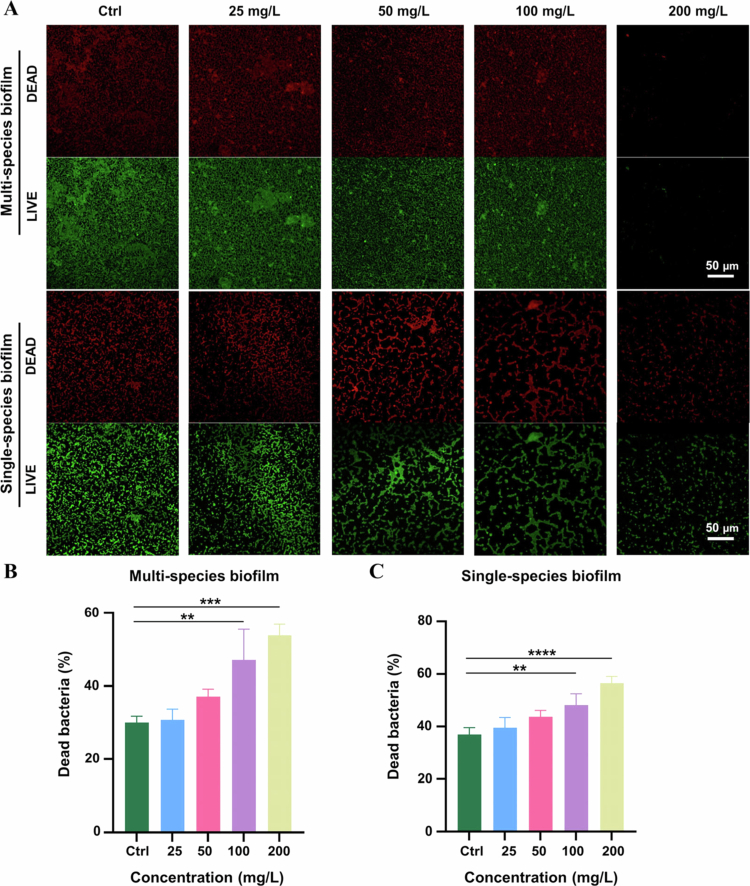
CLSM images of HAS' dispersion on preformed multi- and single-species biofilms treated with different concentrations of HAS. (A) Observation of HAS' effect on biofilms (scale bar = 50 μm). (B) Proportion of dead bacteria after HAS treatment of multi-species biofilms. (C) Proportion of dead bacteria after HAS treatment of *P. gingivalis* single-species biofilms. **p < 0.01, ***p < 0.001 and ****p < 0.0001.

 The impact of HAS on the morphology and bacterial viability within biofilms was further observed *via* CLSM. The results shown in Figure 4 demonstrated significant disruption of biofilms by HAS. HAS-treated biofilms exhibited significant structural alterations, including markedly reduced biofilm density and disintegration of dense bacterial clusters into dispersed fragments, indicating HAS-treated compromise of biofilm integrity. Analysis revealed concentration-dependent shifts in bacterial viability. When the concentration reached 100 mg/L, the proportion of dead bacteria in multi-species biofilms and *P. gingivalis* biofilms increased significantly (Figure 4B,C). These findings suggested that high-concentration HAS induced large-scale bacterial membrane rupture or lethal damage. Collectively, these findings confirmed that HAS exerted robust anti-biofilm effects on both single- and multi-species periodontal pathogenic biofilms, demonstrating its potent efficacy against biofilms.

### The biocompatibility of HAS

**Figure 5.**
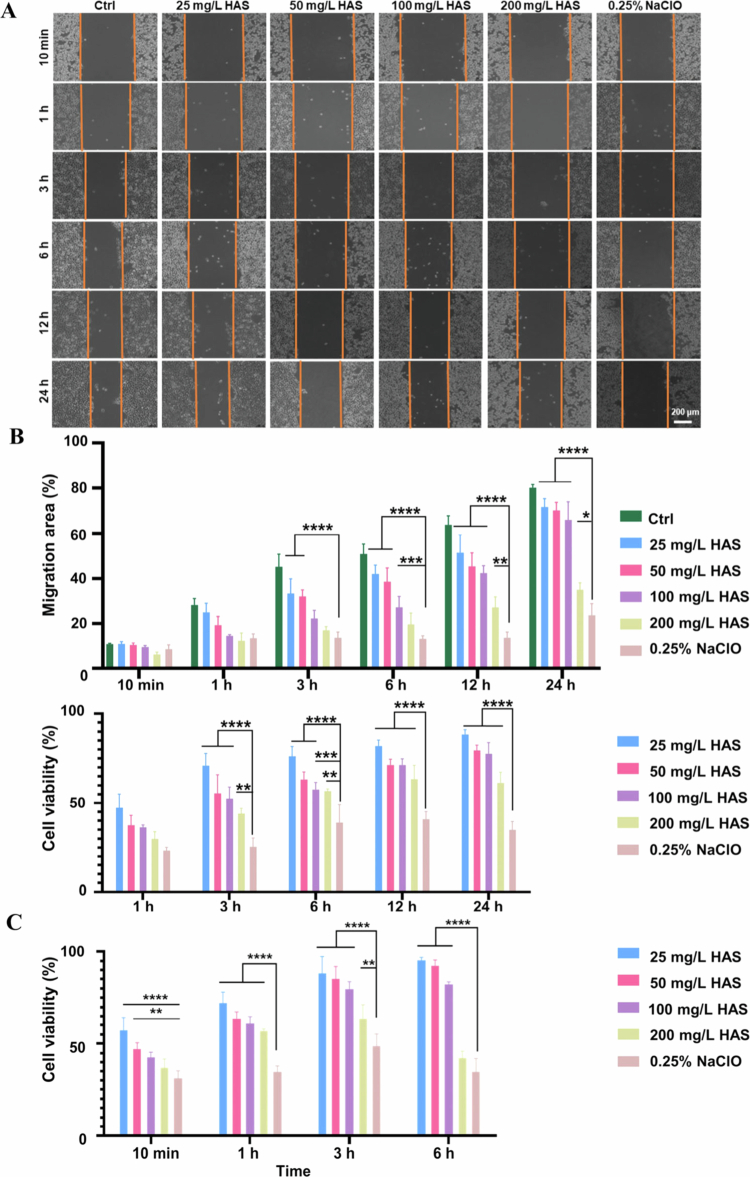
Biocompatibility of HAS. (A and B) The scratch experiment revealed the cell migration of HOK, and CCK-8 assay showed the cell viability of HOK. (C) CCK-8 assay showed the cell viability of hPLFs (scale bar = 200 μm). *p < 0.05, **p < 0.01, ***p < 0.001 and ****p < 0.0001.

The scratch assay was conducted to evaluate the effects of different solutions on the migration capacity of HOK (Figure 5). HOK treated with HAS exhibited a more dynamic morphology at the scratch edge with pronounced pseudopodia extensions (Figure 5A). Analysis revealed that after 24 h of incubation, HOK treated with 100 mg/L HAS achieved a migration rate of 74.58 ± 7.95%, while those treated with 0.25% NaClO solution demonstrated a significantly lower migration rate of 19.54 ± 5.20% (Figure 5B).

The CCK-8 assay was used to evaluate cell viability after treatment with different solutions. The results showed that HOK treated with HAS (77.57 ± 6.30%) exhibited significantly higher viability compared to those exposed to 0.25% NaClO solutions (34.96 ± 4.57%) after 24-h of treatment. In addition, after 6-h of co-culture, hPLFs treated with 100 mg/L HAS retained 83.82 ± 1.33% viability, which was significantly higher than the 0.25% NaClO solution-treated group (43.09 ± 7.39%) (*p* < 0.0001).

### Anti-biofilm mechanism of HAS

#### HAS decreased expression of virulence factors

**Figure 6.**
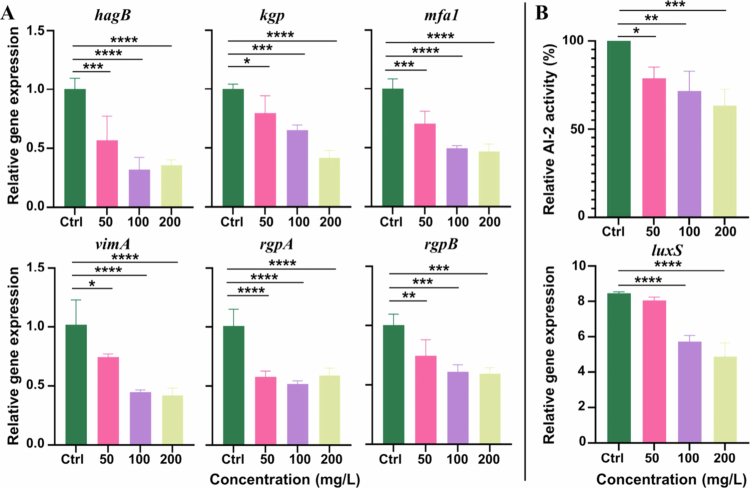
HAS upregulated *P. gingivalis* biofilm virulence factors and inhibited the quorum-sensing system. (A) Expression of virulence factors after HAS treatment. (B) Relative AI-2 activity of *P. gingivalis* biofilms, and the expression of biofilm QS-related gene after HAS treatment. *p < 0.05, **p < 0.01, ***p < 0.001 and ****p < 0.0001.

Compared to the control group, all the examined virulence factors of *P. gingivalis* biofilms were downregulated in a dose-dependent manner (Figure 6A). These genes included *hagB* (involved in hemagglutination); *kgp*, *rgpA*, *rgpB* (involved in gingipain); *vimA* and *mfa1*.

#### HAS inhibited quorum-sensing AI-2 activity

The bioluminescence assay was used to assess the inhibition of HAS on AI-2 activity in *P. gingivalis* biofilms (Figure 6B). Results demonstrated that compared to the control group, 100 mg/L HAS reduced AI-2 activity by 23.91 ± 3.88% (*p* < 0.01). The key gene *luxS* (encoding S-ribosylhomocysteine lyase, which is also involved in biofilm development) of QS treated with 100 mg/L HAS was significantly downregulated (*p* < 0.0001), confirming HAS-mediated disruption of QS signalling at the transcriptional level.

#### HAS induced oxidative stress

**Figure 7.**
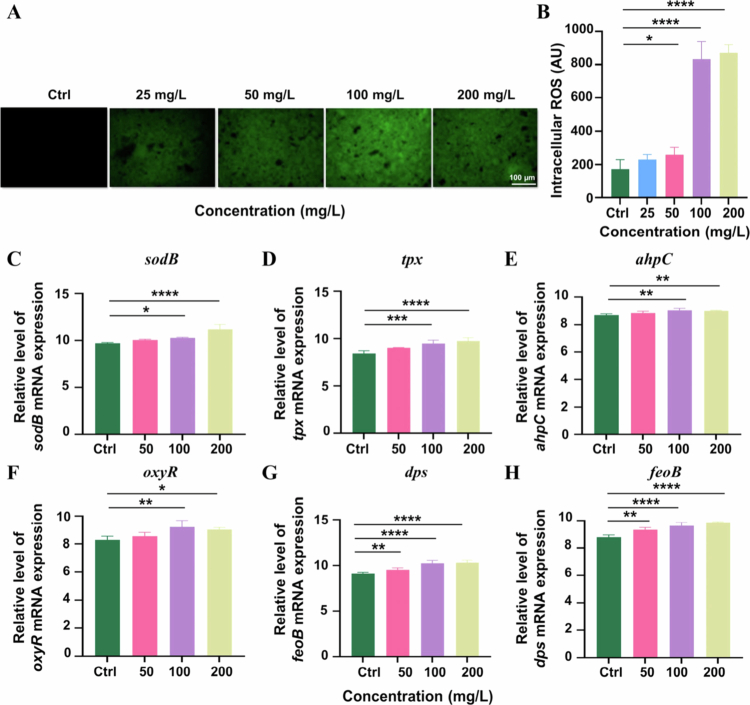
HAS induced oxidative stress in *P. gingivalis* biofilm. (A) ROS fluorescence differences of *P. gingivalis* biofilms (scale bar = 100 μm). (B) Intracellular ROS changes after HAS treatment of *P. gingivalis* biofilms. (C–H) Changes in *P. gingivalis* biofilm ROS-related gene expression after HAS treatment. *p < 0.05, **p < 0.01, ***p < 0.001 and ****p < 0.0001.

The levels of ROS in HAS-treated *P. gingivalis* biofilms significantly increased (Figure 7A,B). Specifically, the ROS levels in the 100 mg/L HAS group were 6.8-fold higher than those in the control group, while no significant difference was observed in the 25 mg/L HAS group. Based on this dose‒response relationship, concentrations of 50−200 mg/L were selected for subsequent gene expression analysis.

Evaluation of ROS-related genes in *P. gingivalis* biofilms demonstrated up-regulation in the HAS-treated groups (Figure 7C–H). The Antioxidant defence genes (*sodB*, *tpx* and *ahpC*) exhibited increased expression in the 100 mg/L HAS group (*p* < 0.05). The redox regulatory genes *oxyR* and *dps* were upregulated in the 100 mg/L HAS group, and the iron metabolism gene *feoB* exhibited increased expression. These results indicate that HAS exerts anti-biofilm effects by inducing oxidative stress in *P. gingivalis* biofilm.

#### HAS reduced bone resorption and inhibited periodontal inflammation *in vivo*

To assess the effects of HAS on bone resorption and periodontal inflammation in rat models, we examined the intensity of the inflammatory infiltrate and cytokine immune reactivity.

**Figure 8.**
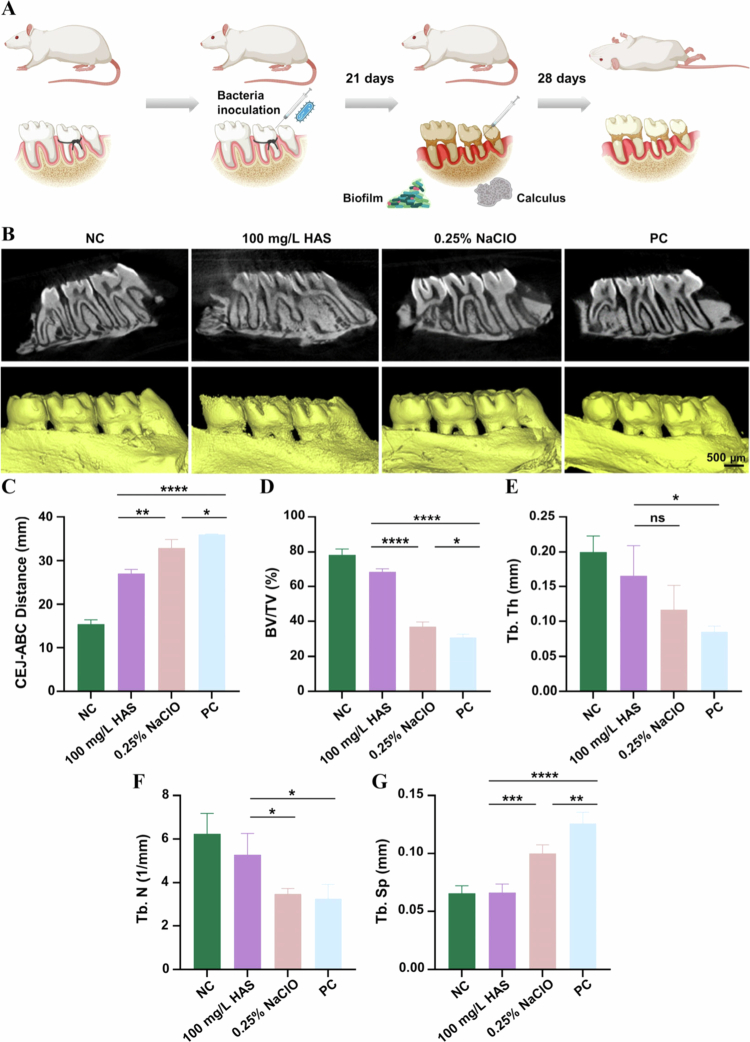
Effect of HAS on ABL in the rat periodontitis model. (A) Flowchart of the animal experiments (scale bar = 500 μm). (B) 2D and 3D reconstruction images of maxillary alveolar bone. (C‒G) Analysis of CEJ–ABC distance, BV/TV, Tb. Th, Tb. N, Tb. Sp. *p < 0.05, **p < 0.01, ***p < 0.001, ****p < 0.0001 and *n*.s. = no significance.

The ligature wires successfully induced bone destruction in rats (Figure 8B). As shown in Figure 8C, the CEJ-ABC distance in the 100 mg/L HAS group was significantly lower than that in the 0.25% NaClO solution group and the PC group, indicating higher bone level. These results demonstrated that the 100 mg/L HAS group exhibited a significantly higher bone volume fraction (BV/TV ratio) compared to both the PC and 0.25% NaClO groups (Figure 8D), indicating superior bone preservation. Micro-architectural analysis revealed that the 100 mg/L HAS treatment significantly increased trabecular thickness (Tb. Th) and trabecular number (Tb. N) in alveolar bone (Figures 8E–F), suggesting that HAS not only prevented bone loss, but also maintained better trabecular microarchitecture than conventional treatments, with the structural parameters approaching those of healthy controls (Figure 8E,F). Additionally, the lower Tb. Sp value in the 100 mg/L HAS group represented reduced bone resorption. All these results demonstrated that 100 mg/L HAS treatment significantly alleviated further alveolar bone absorption in periodontitis.

**Figure 9.**
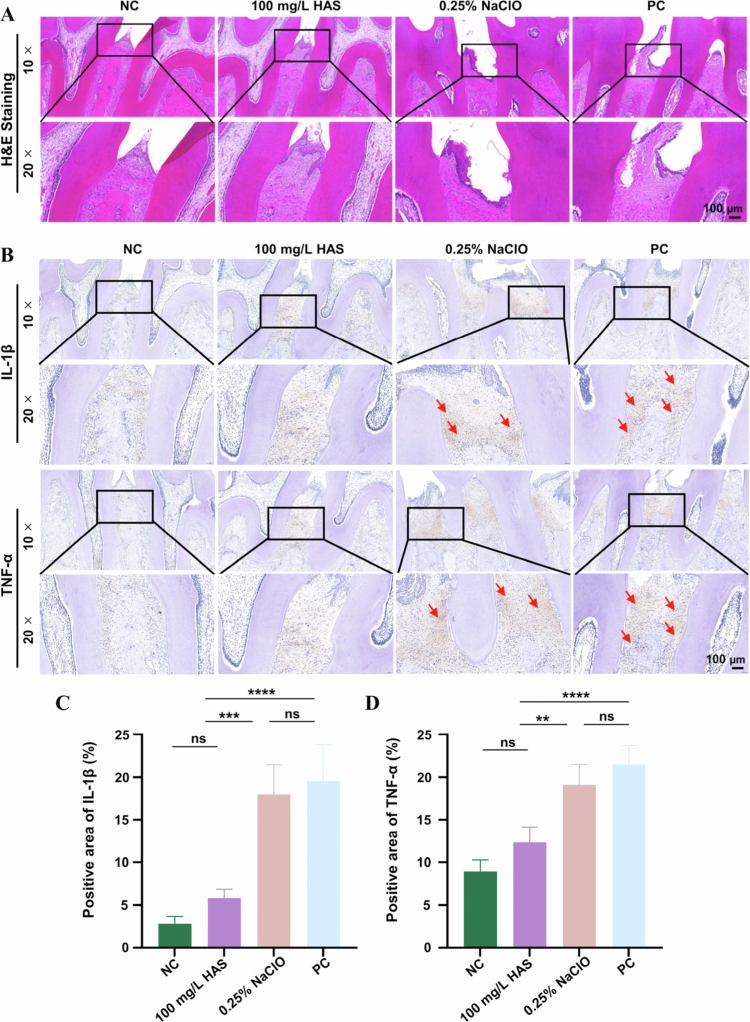
Histomorphological evaluation of inflammation of the periodontal tissue. (A) H&E staining of maxillary tissue section. The black framed area above is the enlarged area, which is shown below. (B) Immunohistochemistry images. The arrows indicate IL-1β-positive cells and TNF-α-positive cells. (C) Quantification of TNF-α and IL-1β. *p < 0.05, **p < 0.01, ***p < 0.001 and ****p < 0.0001.

 H&E staining showed inflammatory infiltration of gingival tissues in the inflammatory group combined with apical migration of the junctional epithelium (Figure 9A). In the PC group, there was significant loss of alveolar bone and extensive necrosis under the alveolar bone. The nuclei of the cells in the necrotic area were pyknotic, fragmented, or lysed, with a large number of inflammatory cells infiltrated. Nevertheless, HAS treatment significantly reduced these pathological processes. The epithelial tissue in HAS resembled normal epithelium, with a regular arrangement of gingival epithelial cells and minimal inflammatory cell infiltration.

In Figure 9B–D, the number of IL-1β- and TNF-α-positive cells markedly increased in the periodontitis group compared to the control group. After treatment, the number of IL-1β- and TNF-α-positive cells decreased compared to the PC group. Notably, the effect of HAS treatment was more significant than PC group and 0.25% NaClO solution group, with results even resembling the NC group. Thus, 100  mg/L HAS exhibited potent anti-biofilm and anti-inflammatory activity, significantly suppressing inflammatory responses in periodontal tissues *in vivo*.

## Discussion

HAS possesses high oxidising capacity and exhibits excellent bactericidal properties. It is biocompatible, less corrosive to metals, and less likely to induce microbial resistance compared to other chlorine-based disinfectants [33,34]. Despite these advantages, HAS has not yet gained prominence in dental treatment, and its specific anti-biofilm mechanism of HAS remains unclear. Thus, we investigated its anti-biofilm effects on periodontal biofilms and the relevant mechanisms.

Our study showed that HAS effectively disrupted multi-species and *P. gingivalis* single-strain biofilms. In the subgingival environment, *P. gingivalis*, a key pathogen of the ‘red complex’, forms polymicrobial symbiotic systems with *F. nucleatum* and *P. intermedia*, significantly enhancing biofilm matrix density and microbial metabolic activity [35]. *E. faecalis*, a Gram-positive bacterium strongly associated with periapical periodontitis, contributes to periodontal pathogenesis through its potent tissue-destructive enzymes (gelatinase and collagenase) and stimulation of osteoclastogenesis, leading to apical tissue destruction and alveolar bone resorption [36]. When *P. gingivalis* coaggregates with other periodontal pathogens to form multi-species biofilms, the amount of biofilm formation increased significantly, and the ultrastructure is more regular and orderly and rich in pores, indicating that the structure of mixed biofilms is more complex [36]. However, current clinical plaque control strategies exhibit limited penetration efficiency against mature multi-species biofilms, while their efficacy evaluations predominantly rely on oversimplified multi-species models that fail to replicate the true profiles of native microbial communities. Based on this, we selected these four species of bacteria to mimic the sub-gingival multi-culture biofilm microenvironment. HAS significantly inhibited the growth of planktonic periodontal pathogenic bacteria. After exposure to HAS, there was a substantial reduction in the amount of biofilms, with a corresponding decrease in the metabolic activity of biofilms as the HAS concentration increased. SEM and CLSM images suggested that the dense structure of HAS-treated biofilms became sparse and porous, with the intercellular connections gradually disappearing and the bacterial surfaces disintegrating, leading to irregular boundaries. These results suggested that HAS had a significant destructive effect on biofilm, and the effect became more pronounced with increasing concentration.

Cytotoxicity remains a long-term concern with different oral preservatives, such as CHX, cetylpyridinium chloride, NaClO antiseptics and alcohol-containing preservatives. As for the biocompatibility, HAS exhibited highly satisfactory performance, as verified by cytotoxicity test and scratch test in this study. Cells treated with HAS had better cell migration ability, covering longer distances and larger area compared to those treated with 0.25% NaClO solution. Although cell viability decreased with increasing concentrations of HAS, the cell survival rate at concentrations of 25−100 mg/L was still significantly higher than that in the 0.25% NaClO group. Therefore, HAS demonstrated better biocompatibility compared to NaClO.

To investigate the anti-biofilm mechanism of HAS, the gene expressions were analysed. Our data showed that all selected virulence factors were downregulated following the addition of HAS. Among them, gene *vimA* has a multifunctional role in several key processes, such as regulating resistance to oxidative stress, acetyl coenzyme A translocation, lipid A synthesis, glycosylation, anchoring of a variety of surface proteins and biofilm formation [37,38]. Gene *hagB* encodes a hemagglutinin molecule pivotal in mediating attachment to host cells, oral colonisation and biofilms [39,40]. Gingival proteases, the main virulence factors of *P. gingivalis*, are categorised into arginine-specific (Rgp, including RgpA and RgpB) and lysine-specific (Kgp) proteases based on substrate specificity. These proteases are essential for nutrient acquisition, biofilm formation and evasion of host defences. *P. gingivalis* has two different types of hyphae, including the Mfa1 hyphae, which are primarily composed of polymers of the Mfa1 protein that facilitate *P. gingivalis*'s binding to synergistic species in oral biofilms. The reduced expression of virulence factors, as detailed above, represents a potential mechanism of HAS' negative regulation of *P. gingivalis* biofilm formation.

Genes involved in genetic regulation, cellular processes and metabolism are expressed at multiple levels by different strains in response to exposure to antibacterial agents. To further investigate the mechanism of HAS's regulation of *P. gingivalis* biofilm, we conducted examinations of both oxidative stress and QS system. There was an increase in intracellular ROS within *P. gingivalis* biofilms after exposure to HAS. ROS, including superoxide, hydrogen peroxide, hydroxyl radicals and other oxygen- or nitrogen-based reactive species, can damage macromolecules and cause extensive cellular damage and cell death [41]. Therefore, we hypothesised that the generation of ROS mediated the HAS-treated bacteria death in *P. gingivalis* biofilms. To confirm this hypothesis, we proceeded to investigate the expression of oxidative stress-related genes, which are crucial in balancing the overproduction of ROS. The six target genes were categorised into three functional groups for analysis under the ‘Defence-Regulation-Metabolism’ framework: (1) The antioxidant defence system, including *sodB*, *tpx* and *ahpC* genes. Gene *sodB* encodes superoxide dismutase (SOD), a key enzyme involved in bacterial oxidative stress response [42,43], *tpx* encodes thioredoxin peroxidase responsible for scavenging H_2_O_2_ [44], and *ahpC* encodes alkyl hydroperoxide reductase, a major intracellular H_2_O_2_ detoxifier; [45] (2) Redox regulatory hub, comprising *oxyR* and *dps* genes, with *oxyR* encoding the oxidative stress regulator OxyR, which orchestrates redox homeostasis and induces catalase expression in bacteria [46], and *dps* encoding Dps-family proteins that protect DNA from peroxide damage *via* DNA binding, sequester free iron to reduce ROS generation and are entirely regulated by OxyR; [47] (3) Iron metabolism and energy homeostasis, represented by *feoB* (encoding ferrous iron transporter), which facilitates Fe²⁺ uptake to sustain Fenton reaction-associated metal ions and redox balance [48].

In recent years, the rapid increase in antibiotic resistance has made it imperative to discover and develop new strategies to combat microbial infections and bacterial diseases. One such promising strategy is to target QS to destroy biofilm. The QS system is essential for regulating biofilm growth patterns and the expression of virulence-associated genes [31]. Previous studies demonstrated that *P. gingivalis* has a LuxS/AI-2 community sensing system. LuxS/AI-2 signalling regulates haem acquisition and protease and stress-related gene expression within *P. gingivalis* [49]. Lucio's study illustrated that neutral electrolytic water reduced the expression of QS genes in the Gram-negative *H. pylori* and reduced biofilm formation [17]. Therefore, we hypothesised that the *P. gingivalis* (Gram-negative) biofilm can be inhibited by the downregulation of QS genes upon exposure to HAS. The effect of HAS on the QS system of *P. gingivalis* was investigated by detecting AI-2 activity *via* bioluminescence analysis of *V. harveyi* BB170. It was found that HAS effectively inhibited AI-2 activity. At the gene level, the expression of the *luxS* gene was significantly downregulated, suggesting that HAS may act as an anti-biofilm agent by targeting QS.

Combined with these results, it can be concluded that HAS treatment of *P. gingivalis* biofilms resulted in decreased expression of virulence factors, upregulation of oxidative stress-related gene expression, decreased activity of AI-2 molecules and downregulation of QS-related gene expression.

 In the *in vivo* experiments, we constructed the rat periodontitis model by wire ligation and intra-gingival inoculation of *P. gingivalis* to simulate the effect of HAS on periodontitis*.* This model allowed us to observe how HAS affects the alveolar bone resorption and inflammation of periodontal tissues in rats. The results showed that the alveolar bone resorption was significantly inhibited, and the levels of inflammatory factors (TNF-α and IL-1β) were reduced after 100  mg/L HAS intervention. It was concluded that HAS, when applied *in vivo*, can reduce plaque accumulation and periodontal inflammation, thus inhibiting alveolar bone resorption. Nevertheless, the results demonstrated that treatment with 0.25% NaClO solution failed to effectively reduce alveolar bone resorption or mitigate inflammatory infiltration in periodontal tissues, which diverges from previously reported findings and is potentially linked to its compromised biosafety [6–8]. Previous studies have shown that acute exposure to high-chlorine agents induces pulmonary inflammation in mice [50], inhaled naturally evaporated gases from 5% NaClO solution exacerbate asthmatic inflammation in rats [51], and chlorine-based compounds aggravate allergic inflammation in murine models by activating the inflammasome signalling pathway [52]. Consequently, it is hypothesised that chlorine exposure may exacerbate localised inflammation in rats, which could counteract the anticipated antibacterial and anti-inflammatory effects of NaClO in periodontitis models. Such antimicrobial agents with suboptimal biosafety profiles require professional local administration and are unsuitable for prolonged intraoral use, as precise and safe intraoral application in rodent models remains technically challenging.

Our results showed that HAS effectively inhibited periodontal biofilms. Notably, we demonstrated for the first time that HAS was able to disrupt *P. gingivalis* biofilms by inducing oxidative stress and especially by targeting the QS system. However, there were several limitations in this study. The limited range of bacteria species tested, without exploring HAS effects across diverse microbial taxa, fails to recapitulate the complexity of the oral poly-microbial environment. *In vivo*, the exclusive reliance on a rat periodontitis model lacks representativeness, and the restricted focus on two inflammation-associated cytokines (TNF-α and IL-1β) overlooks other critical mediators (*e.g.* IL-6) and bone formation markers (*e.g.* BMP-2, OCN). Consequently, comprehensive *in vitro*/*in vivo* studies and clinical evaluations are needed to validate the broad applicability of the HAS. Ongoing efforts are underway to address these gaps in future studies.

In conclusion, this study showed that HAS can significantly inhibit periodontal pathogen biofilms. The anti-biofilm efficacy of HAS can be attributed to its capacity to profoundly affect the biofilm by both inducing oxidative stress and targeting the QS system. Besides, HAS was proven to be safe for HOK and hPLFs and exhibited fewer adverse effects on the mucosal and periodontal tissues of rats. Consequently, both *in vitro* and *in vivo* results suggested that HAS has high biocompatibility and low systemic toxicity and provided compelling evidence that HAS targets the QS system and induces oxidative stress for periodontal anti-biofilm therapy. Hence, we propose the potential application of HAS as an anti-biofilm agent for periodontal adjuvant therapy.

## Acknowledgements

Xuerong Lv and Xiaoqian Wang contributed to conception, design, data acquisition, analysis and interpretation, drafted and critically revised the manuscript; Ke Yan contributed to conception, design, data acquisition, analysis and critically revised the manuscript; Xiang Han contributed to data acquisition, analysis and critically revised the manuscript; Yiyang Yang, Yuzhuo Ma, Yue Wang and Kewei Zhang contributed to data analysis and interpretation and critically revised the manuscript; Feiyang Wang and Chen Yang contributed to data interpretation and critically revised the manuscript. All authors gave final approval and agree to be accountable for all aspects of the work

We thank Dr. Lu Li of the Affiliated Stomatological Hospital of Nanjing Medical University for her help with the guidance on microbiology.

## Supplementary Material

Supplementary material **Appendix Figure 1.** hPLFs primary cell extraction. Operational procedure for extracting hPLFs primary cells and images of primary cells (scale bar = 100 μm).**Appendix Table 1.** Nucleotide sequences of the primers used in this study.
