# Supplementary material for: Hypochlorous acid solution serves as a potential anti-biofilm therapy for periodontitis via targeting quorum sensing of periodontal pathogens
Source: J Oral Microbiol. 2025 Sep 12;17(1):2557959. doi: 10.1080/20002297.2025.2557959 (PMC12434847; doi:10.1080/20002297.2025.2557959)
Supplement: Supplementary material — Appendix Figure 1. hPLFs primary cell extraction. Operational procedure for extracting hPLFs primary cells and images of primary cells (scale bar = 100 μm).Appendix Table 1. Nucleotide sequences of the primers used in this study. [file ZJOM_A_2557959_SM1707.docx]

**Materials and Methods**

**Biocompatibility of HAS**

Human oral keratinocytes (HOK) were provided by the Jiangsu Provincial Key Laboratory of Oral Diseases. Primary human periodontal ligament fibroblasts (hPLFs) were isolated via the tissue explant method: periodontal ligament tissue samples were collected from the middle third of the roots of extracted healthy premolars or molars (11-16 years old). Tissue explants were placed in a 37 °C, 5% CO₂ cell culture incubator for 2-4 h to allow adhesion to the flask surface, followed by gentle inversion of the flask to continue cultivation. Medium replacement was initiated after 5-7 days and subsequently performed every 3 days. Within 1-2 weeks, spindle-shaped cells migrating from the explant periphery were observed microscopically; once cells exhibited a confluent radial growth pattern, they were sub-cultured for subsequent experiments.


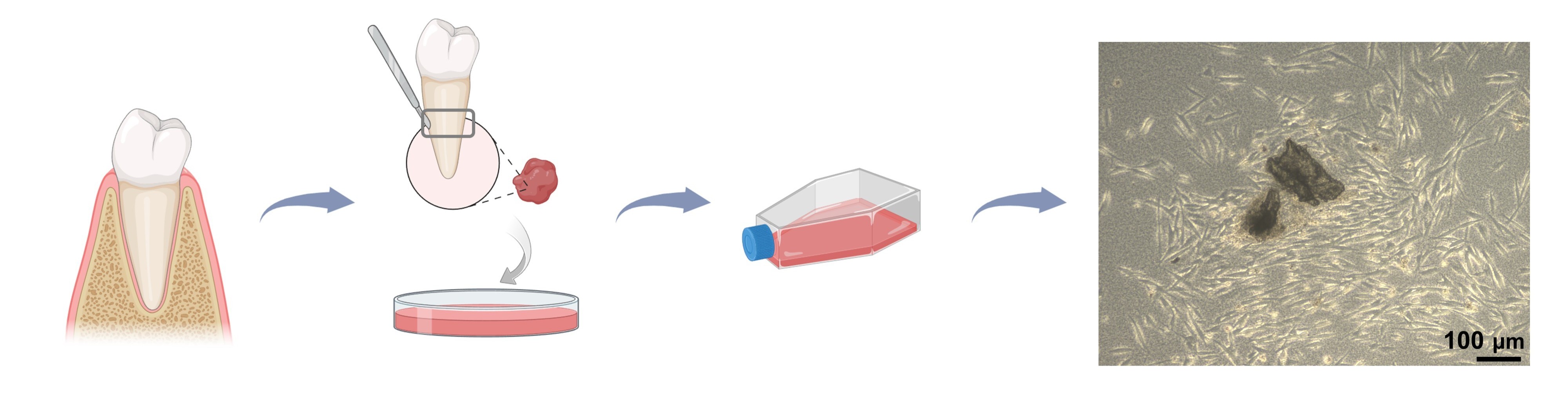


**Appendix Figure 1.** hPLFs primary cell extraction.

Operational procedure for extracting hPLFs primary cells and images of primary cells (scale bar = 100 μm).

The migration test with various concentrations of HAS was conducted using human oral keratinocytes (HOK). Cells (5×10^5^ cells/well) were cultured in a 6-well microplate with Dulbecco’s modified Eagle medium (DMEM; HyClone, Logan, UT, USA) containing 10% fetal bovine serum (FBS; Gibco, Langley, OK, USA) and 1% penicillin/streptomycin solution (Gibco), at a humidified atmosphere of 5% CO_2_ at 37 °C for 1 day to allow attachment. Then, the cells were performed the same grouping and operations as above, and the culture time was 10 min, 1 h, 3 h, 6 h, 12 h, and 24 h. Images were captured by the camera equipped with a 10 × objective and Axio Vision software. Migration rate of cells using ImageJ software. The formula for the calculation of cell migration rate (CMR) is: CMR (%) = (initial scratch area - scratch area after treated) / initial scratch area × 100%.

The cytotoxicity test with various concentrations of HAS was conducted using human periodontal ligament fibroblasts (hPLFs). Cells (5×10^3^ cells/well) were cultured in a 96-well microplate with DMEM containing 5% FBS and 1% penicillin/streptomycin solution, at a humidified atmosphere of 5% CO_2_ at 37 °C for 1 day to allow attachment. Next, the culture medium was replaced with DMEM containing test reagents for the culture time of 10 min, 1 h, 3 h, and 6 h. 0.25% NaClO solution was used as a positive control. The cytotoxicity was determined by using the cell counting kit-8 (CCK-8, Beyotime, Shanghai, China) assay. Each well was added with 10 μL CCK-8. After incubation in the CO_2_ incubator for 1 h to 1.5 h, absorbance was measured at the wavelength of 450 nm. The formula for the calculation of relative growth rate (RGR) is: RGR (%) = (A450 of test group - A450 of blank control) / (A450 of negative control - A450 of blank control) × 100%.

**Appendix Table 1.** Nucleotide sequences of primers used in this study.

| Gene | Description | Primer sequence (5’-3’) | |
| --- | --- | --- | --- |
|  |  | Forward | Reverse |
| *16S rRNA* | normalizing internal standard | TGTAGATGACTGATGGTGAAA | ACTGTTAGCAACTACCGATGT |
| *hagB* | hemagglutinin protein HagB | TGTCGCACGGCAAATATCGCTAAAC | CTGGCTGTCCTCGTCGAAAGCATAC |
| *kgp* | lysine-specifc cysteine proteinase Kgp | AGGAACGACAAACGCCTCTA | GTCACCAACCAAAGCCAAGA |
| *rgpA* | arginine-specifc cysteine proteinase RgpA | CACCGAAGTTCAAACCCCTA | GAGGGTGCAATCAGGACATT |
| *rgpB* | arginine-specifc cysteine proteinase RgpB | GCTCGGTCAGGCTCTTTGTA | GGGTAAGCAGATTGGCGATT |
| *vimA* | virulence modulating gene A | TCGCGTAGTCTGAGAGTAACCTT | GGTATAAACGAAGACAGCACGAC |
| *mfa1* | minor fimbrial antigen Mfa1 | ACTTCTCCCGATTCATGGTG | GGATTCGGGTCAGGGTTATT |
| *luxS* | S-ribosylhomocysteine lyase | GAATGAAAGAGCCCAATCG | GTAATCGCCTCGCATCAG |
| *sodB* | superoxide dismutase | TACAAAAGAGCGAAGGCGGT | ATAGCTTCCCCCAGTTTGCC |
| *feoB* | ferrous iron transport protein B | CGAGTGGAAGCCGACAAGAT | GGCACTATGACCTTCCAGCA |
| *ahpC* | alkyl hydroperoxide reductase, C subunit | GGTGTGACGGTAGAAGCGAA | CTTTGAGCTGGAGGGTCAGG |
| *dps* | Dps family protein | CGACACCGAGCTACAACTGA | AGCATTCCTGCCTTCGTCAA |
| *oxyR* | redox-sensitive transcriptional activator OxyR | CTGCCTCGTGTCTTCCCAAT | GCGCGATACATACCCCAGAA |
| *tpx* | thiol peroxidase | CGCCGTTTCAATCAGGAAGC | TCAAAGGGCCATCGGTCATC |
